# Effect of Microwave Plasma Pre-Treatment on Cotton Cellulose Dissolution

**DOI:** 10.3390/molecules27207007

**Published:** 2022-10-18

**Authors:** Shaida S. Rumi, Sumedha Liyanage, Julia L. Shamshina, Noureddine Abidi

**Affiliations:** Department of Plant and Soil Science, Fiber and Biopolymer Research Institute, Texas Tech University, Lubbock, TX 79409, USA

**Keywords:** cotton fiber, cellulose, oxygen plasma, molecular weight, surface modification

## Abstract

The utilization of cellulose to its full potential is constrained by its recalcitrance to dissolution resulting from the rigidity of polymeric chains, high crystallinity, high molecular weight, and extensive intra- and intermolecular hydrogen bonding network. Therefore, pretreatment of cellulose is usually considered as a step that can help facilitate its dissolution. We investigated the use of microwave oxygen plasma as a pre-treatment strategy to enhance the dissolution of cotton fibers in aqueous NaOH/Urea solution, which is considered to be a greener solvent system compared to others. Attenuated Total Reflectance Fourier Transform Infrared Spectroscopy, Scanning Electron Microscopy, and Powder X-ray Diffraction analyses revealed that plasma pretreatment of cotton cellulose leads to physicochemical changes of cotton fibers. Pretreatment of cotton cellulose with oxygen plasma for 20 and 40 min resulted in the reduction of the molecular weight of cellulose by 36% and 60% and crystallinity by 16% and 25%, respectively. This reduction in molecular weight and crystallinity led to a 34% and 68% increase in the dissolution of 1% (*w*/*v*) cotton cellulose in NaOH/Urea solvent system. Thus, treating cotton cellulose with microwave oxygen plasma alters its physicochemical properties and enhanced its dissolution.

## 1. Introduction

Cellulose, the most abundant biopolymer on Earth, is biosynthesized mainly by plants, algae, and tunicates [1]. Owing to its renewability, abundance, biocompatibility, and biodegradability, it has become a polymer of choice for the preparation of numerous materials commonly produced from petrochemical-based synthetic plastics [2,3,4,5]. Cellulose is a polydisperse linear chain polymer, consisting of anhydroglucose units linked by *β*-glycosidic bonds in the thermodynamically preferred ^4^C_1_ conformation [6]. The abundance of hydroxyl groups in the anhydroglucose units leads to structural complexity and rigid supramolecular structure of cellulose as these hydroxyl groups create numerous intra- and intermolecular hydrogen bonds between cellulosic chains.

Depending on the sources, including cotton, wood, and algae, the degree of polymerization (DP) of cellulose ranges from 300 to 20,000 [7,8]. Compared to cellulose from other sources, cotton cellulose has a higher molecular weight (MW) and a higher level of crystallinity [8]. Inherent polydispersity (expressed through polydispersity index, PDI) greatly depends on the source of cellulose [9]. PDI of cellulose dissolved in a solvent media is a strong indicator of how processable the cellulose solution will be [10] and significantly affect the properties of resulting cellulose-based materials [11]. Highly organized crystalline domains coexist in cellulose with less ordered non-crystalline amorphous domains [12]. In solution, the cellulose chains adopt semi-flexible conformations [13,14,15].

Although quite a few solvent systems have been developed [16,17,18], the potential applications of cellulose are hindered due to its recalcitrance to dissolution in common solvents that result mainly from the aforementioned hydrogen bonding network, high MW, high crystallinity of the polymer, and rigidity of the polymeric chains [19]. “Green conversion” of cellulose to bioproducts is still challenging, and the industry often relies on costly and/or environmentally unsafe solvents.

The dissolution of cellulose requires disassembling the supramolecular structure of cellulose and separating individual chains, or rather bundles of cellulose chains, with minimal breakage of the glycosidic bonds [1]. Traditional volatile organic solvents are unsuitable for dissolving cellulose [20], while *N*,*N*-dimethylacetamide/lithium chloride (DMAc/LiCl), *N*-methylmorpholine-*N*-oxide (NMMO), aqueous alkali solutions, and ionic liquids remain the most effective non-derivatizing solvent systems for cellulose [16,17,18,21]. However, the degree of cellulose dissolution depends on the solvent and is greatly impacted by the physicochemical parameters of cellulose, such as MW and crystallinity [1,22]. Improvement of cellulose dissolution is sought through different pre-treatments to reduce the particle size and facilitate solvent diffusion between cellulosic chains [23], reduce the MW to enhance the entropic driving force of dissolution [24], and reduce the crystallinity (i.e., facilitate amorphization) to increase the effective surface area [25].

Several pretreatment techniques, such as ball milling, cryogrinding, freeze-drying, acidic pretreatment, solvent exchange, hydrothermal treatments, and steam explosion have been reported to facilitate cellulose dissolution by enhancing the accessibility of solvents inside the polymeric network of cellulose [25,26,27,28,29,30,31,32,33]. Pretreatments add an extra step to the conversion process of cellulose into usable products and therefore, increase the processing cost and time and, in many cases, generate extra wastes. Specifically, ball milling, a mechanochemical treatment, widely used to reduce the particle size and MW and increase the effective surface area of the material by amorphization, is an energy-intensive technique [34,35]. There are disparities in ball milling efficiency, especially on its effectiveness. While in some cases ball milling leads to a remarkable decrease in MW in a short period of time, in other cases it hardly reduces the MW even after a prolonged milling. For example, Shrotri et al. reported a 25% reduction in MW of cellulose after 5 h of ball milling [35], whereas Ling et al. demonstrated a 75% reduction in MW after 2 h of ball milling [36]. In addition, although ball milling treatment was shown to be effective in amorphization of cellulose, it does not always lead to improvement in dissolution. Thus, Ishii et al. reported that even though the crystalline structure of the ball-milled cellulose was almost destroyed, the dissolution of cellulose was not significantly improved [33]. However, the authors found that the solvent exchange with DMAc, which did not significantly change the crystallinity or the degree of polymerization (DP) of cellulose, had a remarkable positive effect on cellulose dissolution, under the same dissolution conditions. 

Similar to ball milling, acid hydrolysis leads to a significant depolymerization of cellulose chains [37,38], thereby facilitating the dissolution process. Yet, in some instances, the impact of acid hydrolysis on enhancing the dissolution of cellulose is not significant. One study reported that acid hydrolyzed cellulose showed an insignificant improvement in its dissolution, although the MW of cellulose decreased by ten folds [6]. It is likely because acid hydrolysis depolymerizes cellulose chains in amorphous regions leaving behind highly crystalline cellulose that is hard to dissolve. Moreover, acid hydrolysis requires a large volume of water for the neutralization process and generates a significant volume of acidic waste [39]. Hydrothermal and steam explosion pretreatments have also been shown to successfully improve the dissolution of cellulose by reducing the DP, but they require high-pressure and high-temperature systems [31,32].

Plasma, a gaseous mixture of charged particles, such as free electrons, photons, ions, radicals, and metastable molecules, interacts with material surface and reactively alters its physicochemical properties. Plasma treatment facilitates either removal or addition of particles from/to the surface by different types of reactions (including surface cleaning, etching, grafting, polymerization, and radicalization) [40,41,42,43,44], and stands out as a promising approach to modify the physicochemical properties of cellulose [45]. It has substantial advantages over the aforementioned pretreatment methods in terms of cost, time, and energy [46]. Moreover, unlike wet-chemical pretreatments, plasma treatment offers a greener and more ecofriendly route to cellulose dissolution as it does not generate chemical wastes [47], although the scalability of the technology is a challenge in its applicability to industrial processes.

A limited number of studies reported the effect of plasma pretreatment on the physicochemical properties of cellulose extracted from different sources (e.g., microcrystalline cellulose, cotton linter cellulose powder, cotton pulp, sugarcane bagasse pulp, and luffa/sponge gourd), and its direct impact on the cellulose dissolution in various solvent systems, including DMAc/LiCl [28], sodium hydroxide [48], a mixture of 1-butyl-3-methylimidazolium acetate ([C_4_mim][OAc]) ionic liquid and dimethyl sulfoxide (DMSO) [49], and anhydrous phosphoric/polyphosphoric acid solvent system [50].

Cotton cellulose is characterized by high MW and crystallinity, and NaOH/urea solvent system is considered a greener solvent system compared to many other existing solvents [19]. Considering the limited literature on plasma pretreatment of high MW cellulose to enhance its dissolution, we investigated the effect of microwave oxygen plasma on cotton cellulose dissolution. In this study, cotton fibers were first treated with oxygen plasma and then subjected to dissolution in NaOH/urea solvent system.

## 2. Results and Discussion

### 2.1. Dissolution of Cotton Fiber in Aqueous NaOH/Urea Solvent System

Both untreated and plasma surface-modified (PSM) cotton fibers were dissolved in aqueous NaOH/urea and respective solutions were analyzed under cross-polarized light. Cotton fiber is strongly birefringent, and the crystalline areas of cellulose in cotton fibers show greater birefringence and appear brighter in PLM images [8]. 

**Figure 1:**
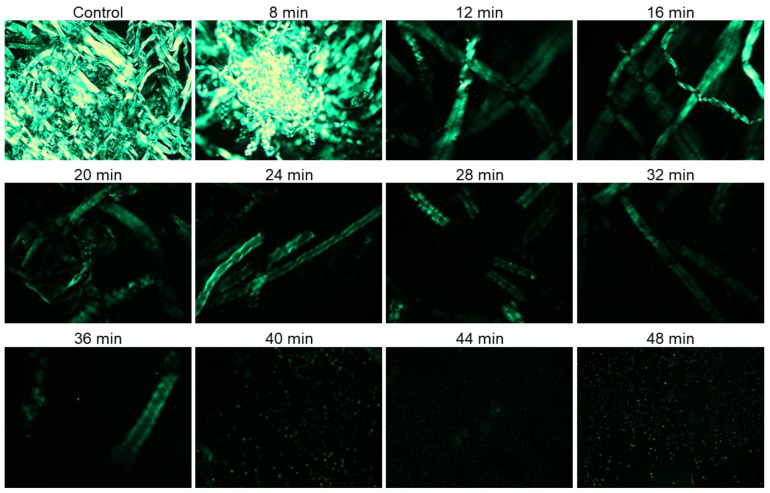
Polarized light microscopy images of 1% (*w*/*v*) cotton fibers/NaOH/urea solution after 30 min of dissolution in ambient temperature (magnification ×10): Cotton fibers were exposed to oxygen microwave plasma for different period of time.

During dissolution, the solvent penetrates, swells, and gradually destroys the polymer crystalline domains. Hence, PLM could be used to monitor the effect of plasma pretreatment on the dissolution of cotton fibers. Specifically, the gradual disappearance of birefringence as a function of plasma exposure time was taken as an indication of cotton fiber dissolution. Figure 1 shows PLM images of PSM cotton fibers dissolved in aqueous NaOH/urea solvent system for 30 min. Rapid dissolution of cellulose in NaOH/Urea solvent media (2 min) has been reported [51] with the cellulose polymer of significantly lower DP than cotton cellulose. In our case, a relatively prolonged time of 30 min was determined to be necessary because shorter, 10, and even 20 min of dissolution time was insufficient for homogenous dispersion of fibers, whereas 30 min of dissolution time under stirring helped disperse the cotton fibers in the solvent and produced a homogenous solution. It is apparent that cotton fiber dissolution was significantly improved with the increase of the plasma exposure time.

A large number of intact cotton fibers was observed in the images collected from the solution of untreated cotton fibers in aqueous NaOH/urea system. These fibers appeared highly crystalline, intact, and exhibited a ribbon-like conformation of cellulose. No major changes were noticed in the PLM images of cotton fibers exposed to plasma for 8 min, and a highly aggregated network of cotton fibers was still present. The birefringence, however, gradually reduced with further increase of plasma-treatment time, and starting from 12 min treatment, important changes in cellulose dissolution were detected. Thus, highly entangled fibrous structures were no longer observed, and the remaining fibers appeared swollen and exhibited a helical structure of cellulose microfibrils. This phenomenon might be related to faster disappearance of crystalline areas associated with improved cellulose dissolution. The fragments of cotton fibers were seen in the solution of PSM-24. Fewer fiber fragments were detected with further increase in plasma treatment time, and only a trace amount of highly fragmented undissolved fiber particles was visible in the PSM-40 solution. However, further improvement of cellulose dissolution was not achieved when cotton fibers were plasma-treated for 44 and 48 min.

According to polarized light microscopy analysis, the domain of anisotropic cellulose gradually drops down with the increase in plasma treatment time, indicating that plasma treatment is highly conducive to enhancing cellulose dissolution. This is likely associated with one or more physicochemical changes in cotton cellulose caused by energetic particles of oxygen plasma. Based on these results, PSM-20 and PSM-40 were selected for further analysis of the effect of plasma treatment on the morphological and physicochemical properties of cotton fibers that were responsible for their improved dissolution.

**Figure 2:**
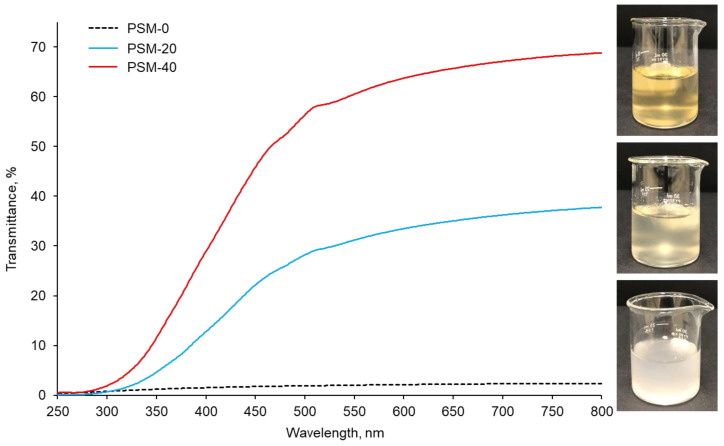
UV-vis light transmittance collected from the control and plasma treated cotton fibers (PSM-0 (control), PSM-20, and PSM-40) dissolved in NaOH/urea solvent system for 30 min (1% *w*/*v*). Inset shows the corresponding visual images of cellulose/NaOH/urea solutions.

In addition to PLM investigations, cellulose dissolution was also monitored by light transmittance data collected using UV-Vis spectroscopy, which measures the intensity of the light beam going through the sample. The UV-Vis spectra of PSM-0 (control), PSM-20, and PSM-40 cellulose solutions are shown in Figure 2. The PSM-0 solution showed a turbid appearance due to suspended undissolved cotton fibers. This resulted in low intensity of direct transmitted light, due to the light scattering by the undissolved cotton fiber particles, accounting for approximately 2% at 800 nm. In contrast, the PSM-20 solution looked clearer compared to the control PSM-0 solution suggesting improved fiber dissolution. The intensity of direct transmitted light significantly increased in the PSM-20 solution accounting for approximately 41% of transmittance at 800 nm. PSM-40 solution exhibited the highest transmittance of 64%, which indicated enhancement of the dissolution of cellulose. According to statistical analysis, light transmittance significantly increased with the plasma treatment time (*n* = 27 and *p* ≤ 0.05).

### 2.2. Effect of Plasma Treatment on Molecular Weight (MW) and Molecular Weight Distribution (MWD) of Cotton Fiber Cellulose

**Figure 3:**
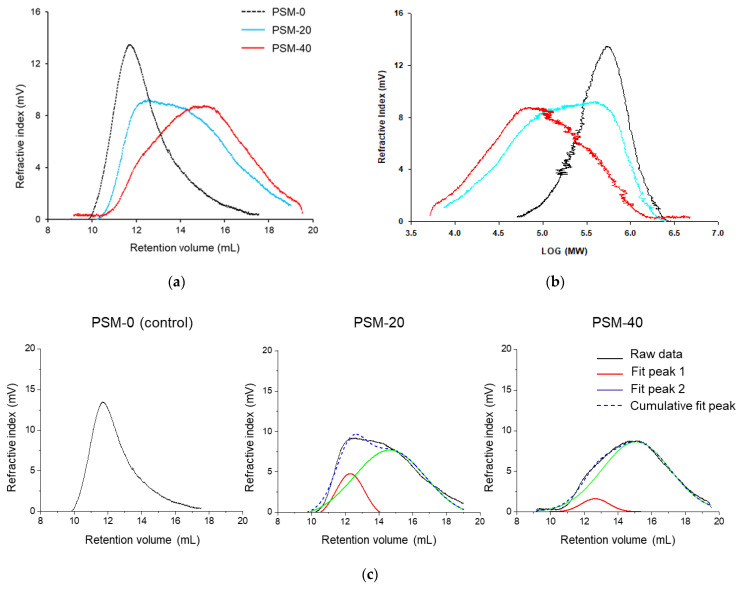
Representative gel permeation chromatography (GPC) data of the control and plasma surface modified (PSM) cotton cellulose. (**a**) Refractive index vs. retention volume, (**b**) Refractive index vs. log MW of cotton cellulose subjected to plasma treatment for 0 min (PSM-0), 20 min (PSM-20), and 40 min (PSM-40) and, (**c**) Peak deconvolution of refractive index vs. retention volume curves of PSM-0, PSM-20, and PSM-40 cotton cellulose.

Gel permeation chromatography (GPC, a type of Size Exclusion Chromatography, SEC) was performed to study the effect of plasma exposure time on MW and MWD of cotton fiber cellulose. As it could be seen in Figure 3a,b and Table 1, PSM-0 cellulose (control) exhibited higher MW and narrower MWD compared to cellulose in PSM cotton fibers. The peak retention volume appeared to increase as a function of plasma treatment time, indicating that PSM samples were of lower MW. This implied that depolymerization of large cellulose macromolecules into smaller chains was induced by plasma treatment. 

Indeed, the MW of untreated cotton cellulose was determined to be 3.9 × 10^5^ Da, and plasma pretreatment resulted in a significant reduction of MW of cellulose (*p* ≤ 0.05). Plasma pretreatment of cotton fibers for 20 and 40 min decreased the MW of cellulose by 36% (2.5 × 10^5^ Da) and 60% (1.5 × 10^5^ Da), respectively. Shepherd et al. reported a similar effect of oxygen plasma treatment on the MW of cellulose [52]. The authors found that the initial level of polymer degradation was largely dependent on the level of impurities, such as wax substances, located on the sample surface. Such reduction of the MW could lead to a better dissolution of cellulose in NaOH/urea solvent system.

It was also observed that peaks exhibited broader peak widths compared to the control with the increase in plasma treatment time, implying wide MWD of the PSM-treated samples. Moreover, it appeared that MWD peaks of PSM-treated samples consisted of two overlapping peaks, resulting in a bi-modal distribution. To further understand this, raw GPC data was exported to OriginPro software and peak deconvolution was performed (Figure 3c), assuming Gaussian distribution, with symmetry around the highest point. 

It was determined that for the control sample, the refractive index peaked around 12 mL of retention volume. Plasma treatment for 20 min resulted in a decrease in the intensity of this peak, and additional plasma treatment for 20 more min further subsided the intensity of this peak, decreasing the amount of high MW cellulose with the increase of the plasma treatment time. A simultaneous presence of another peak was also observed in PSM-20 samples around 15 mL of retention volume, suggesting the presence of low MW cellulose; the intensity of this peak significantly increased after 40 min plasma treatment.

PDI (shown in Table 1) is a sensitive measure of polymer’s degradation. Cellulose processing for material preparation is significantly impacted by its PDI value [10]. The calculated PDI value for untreated fibers was found to be 2.09 ± 0.83. The PDI significantly increased after 20 min plasma treatment time, to 7.10 ± 5.44, indicating broad MWD. Such significantly higher PDI could be explained by increased low MW cellulose fraction resulting from random cellulose chain breakage, in addition to thesignificant proportion of long polymer chains. In other words, this PDI value arises from contributions of two peaks (Figure 3c). The PDI value decreased with the increase of plasma treatment duration to 40 min, and was determined to be 3.82 ± 2.00, indicating breakage of long chains, and a further significant increase of low MW cellulose fraction.

### 2.3. Effect of Plasma Pretreatment on Surface Morphology of Cotton Fibers

**Figure 4:**
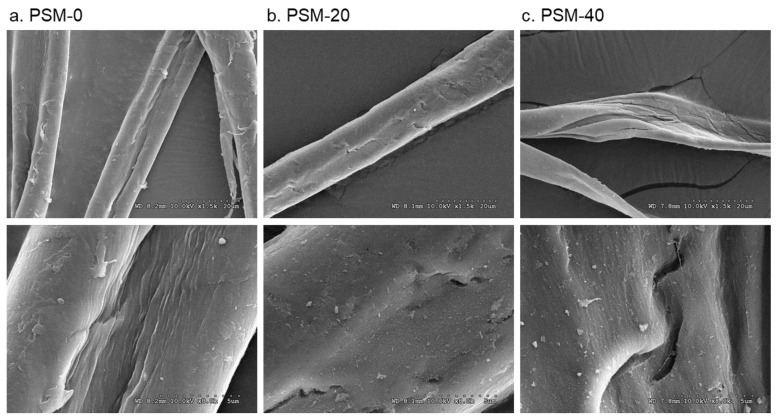
Effect of plasma pretreatment on the surface morphology of cotton fibers. SEM micrographs of (**a**) control cotton fibers (PSM-0) and plasma surface modified fibers for (**b**) 20 min (PSM-20) and (**c**) 40 min (PSM-40). (Magnification: ×1.5K (**top**) and ×8K (**bottom**)).

The surface morphology of the control and plasma-treated cotton fibers is shown in Figure 4. The change in surface morphology of cotton fibers with oxygen plasma treatment was observed. Thus, the surface of untreated cotton fibers appeared smooth and even, whereas the morphology of PSM fibers looked altered with clear pitting and splitting of the fiber. Fibers that were plasma-treated for 20 min exhibited a rough surface and noticeable fractures. These changes looked prominent in fibers treated for 40 min. This is likely attributed to the etching effect of highly energized plasma particles. Similar physical degradation was reported in several studies when fibers were exposed to oxygen plasma as well as DC air plasma and di-electric barrier discharge plasma (DBD) [49,52,53,54]. The presence of surface cracks and defects in plasma-pretreated cotton fibers could improve cellulose dissolution as they can facilitate solvent diffusion and accessibility to the internal well-organized structures of cellulose.

### 2.4. Effect of Plasma Pretreatment on Surface Morphology of Cellulose Films

**Figure 5:**
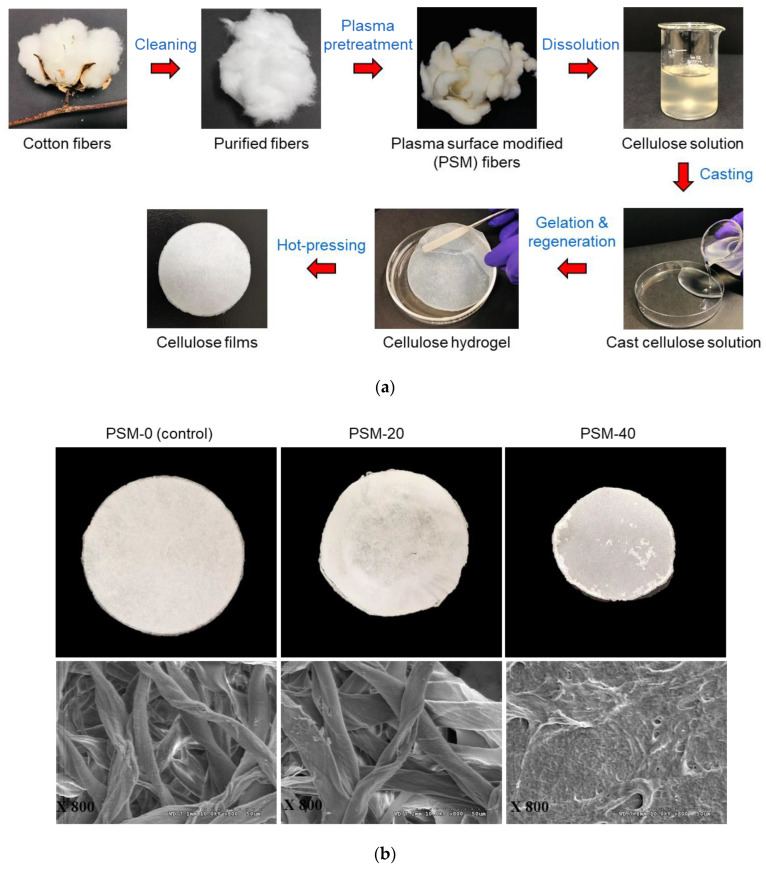
Preparation of cellulose films from plasma surface modified (PSM) cotton fibers and their morphological characterization. (**a**) Preparation of cellulose films through casting, gelation, regeneration, and hot-pressing and (**b**) Visual images (**top**) and SEM micrographs (**bottom**) (magnification: ×800) of regenerated cellulose films prepared from PSM cotton fibers (PSM-0 (control), PSM-20, and PSM-40).

The preparation steps of cellulose films from cellulose solutions through casting, gelation, regeneration, and hot pressing are summarized in Figure 5, that shows visual images and SEM micrographs of films prepared from the control and PSM cotton fibers. The control film looked similar to a filter paper, and the SEM micrographs showed highly twisted intact cotton fibers arranged in a highly aggregated network. It reveals a poor solubility of untreated cotton fibers in NaOH/Urea solvent system. The film prepared from PSM-20 cotton fibers looked similar to PSM-0, and SEM micrographs confirmed the presence of intact cotton fibers entangled together forming a dense network. However, cotton fibers looked somewhat swollen and less twisted compared to that of the control fibers, possibly due to improved solvent diffusion into the internal structures of cotton fibers. Indeed, oxygen plasma pretreatment of intact cotton fibers for 20 min is not sufficient enough to fully disintegrate cotton fibers in NaOH/urea solvent system.

Regenerated cellulose film prepared from PSM-40 cotton fiber presented a densely packed surface structure with no presence of undissolved intact fibers indicating that PSM-40 cotton fibers were completely dissolved in NaOH/urea solvent system. However, the surface of the film did not look smooth. Similar morphological properties have been reported in the regenerated cellulose film prepared from plasma-treated luffa cellulose [50].

### 2.5. Attenuated Total Reflectance (ATR) Fourier Transform Infrared (FTIR) Spectroscopy Analysis of Control and PSM Cotton Fibers

**Figure 6:**
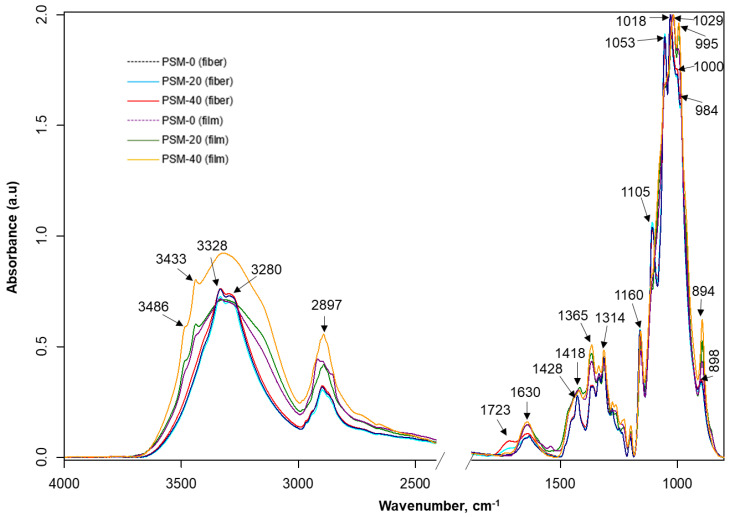
FTIR analysis of plasma surface modified cotton fibers and their corresponding regenerated films. FTIR spectra of control (PSM-0) and oxygen plasma surface modified (PSM-20 and PSM-40) cotton fibers and regenerated cellulose films prepared from control and PSM cotton fibers.

**Figure 7:**
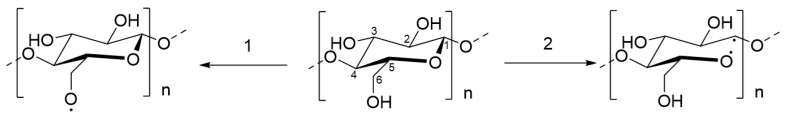
Radical formation on cellulose molecules upon exposure to oxygen plasma: (1) dehydrogenation at C6 and (2) cleavage of pyranosidic bond (O(ring)-C1).

The FTIR spectra of untreated and PSM cotton fibers are shown in Figure 6. Untreated cotton fibers present all characteristic vibrations of native cellulose [44]. Although the FTIR spectra of untreated and PSM cotton appear nearly identical, there are some noticeable changes. Thus, in PSM cotton fibers, a new infrared vibration emerged at ~1723 cm^−1^ that is usually attributed to -C=O stretching in -CHO/-COOH [55]. Given the aforementioned premise of the reduction in the MW, this observation can be interpreted by the degradation of cellulose in accordance with two mechanistic pathways reported by Vaideki et al. [55]. Accordingly, the presence of the carbonyl group could be attributed to the homolytic cleavage of the OH group on C6 carbon atom with a radical formation, and its subsequent oxidation with a formation of the carbonyl C(O) group (Figure 7, pathway 1). Another possibility is the homolytic breakage of the pyranosidic bond between O(ring)-C1 also with a formation of oxygen radical, and subsequent oxidation [55] (d, pathway 2). Meanwhile, it was also noticed that the intensity of the vibration ~1160 cm^−1^, attributed to the stretching mode of C-O-C (pyranose ring), is slightly decreased with the increase in plasma exposure time [56]. This supports the breakage of the pyranosidic bond between O(ring)-C1. Other studies also reported pyranosidic cleavage of cellulose chains forming -C=O group due to the exposure of cellulose to oxygen plasma [42,52,56]. In addition, the products of oxidation reactions might increase the intensity of -C=O group as well. Overall, these observations suggest depolymerization of cellulose, supporting GPC analysis results. 

Additionally, there are nominal changes (e.g., peak shifting and slight changes in peak intensities) in the carbohydrate fingerprint regions (~1105, 1053, 1000, and 984 cm^−1^: associated with C-O stretching of cellulose) emphasizing a slight disturbance in the cellulose backbone. However, there is hardly any change in the vibrations around 1029 cm^−1^ and 1205 cm^−1^, which are also attributed to C-O stretching of cellulose [57]. Therefore, it can be inferred that plasma treatment changes the surface chemistry of cellulose by depolymerizing and oxidizing long polymer chains that result in carbonyl formation. 

It is well-known that some infrared bands of cellulose are sensitive to changes in crystalline structures (e.g., ~897 cm^−1^-*β*-linkage of cellulose and ~1428 cm^−1^: CH_2_ scissoring) [25]. The infrared spectra of plasma-treated cotton fibers do not show major changes in the intensity of 897 cm^−1^, although the intensity of the crystalline absorption band 1428 cm^−1^ shows a negative trend with the increase of plasma exposure time. 

The infrared spectra of regenerated cellulose films prepared from the untreated (control) and treated (PSM-20 and PSM-40) cotton fibers are also shown in Figure 6. The FTIR spectra of regenerated cellulose represent distinctive infrared bands of cellulose II, suggesting the transformation of cellulose I during the dissolution and regeneration steps [58]. While these spectra appear similar, they have certain distinctions from each other.

Notably, after regeneration, vibrations 3487 and 3442 cm^−1^ that are attributed to intramolecular hydrogen-bonded O-H, appeared when compared to non-regenerated cellulose fiber. These vibrations appear to be small in the film prepared from untreated fibers, strong in the film prepared from PSM-20 fibers, and most pronounced in the film prepared from PSM-40 fibers. Intermolecular hydrogen-bonded O-H was seen at lower wavenumbers (3338 and 3296 cm^−1^) in the film prepared from untreated fibers, while in PSM-20 and PSM-40 film, these appeared as a single peak at 3320 cm^−1^. This implies hydrogen bonds reorientation and van der Waals forces weakening between the original cellulosic chains, typical for conversion of cellulose I into cellulose II [59], and re-formation of intramolecular hydrogen-bonding network. Another noticeable change is the disappearance of earlier detected carbonyl stretch in fiber upon preparation of PSM-20 and PSM-40 films. The proposed reason for this is that the formation of carbonyl C=O in PSM-20 and PSM-40 fibers took place at the fiber surface, and was easily detectable by ATR. After complete dissolution and regeneration, original cellulosic chains reoriented, causing the carbonyl groups being evenly distributed throughout the films, with lower and not easily detectable concentration at the surface. The differences were also detected in C-O-C (anti-symmetrical bridge stretching vibration), located at 1157 cm^−1^, due to ring degradation induced by plasma. The change in the peak at 894 cm^−1^ attributed to *β*-linkage of cellulose was also detected. No other significant changes were noted supporting already known fact that the dissolution of cellulose in NaOH/urea solvent system does not cause derivatization of cellulose.

### 2.6. Effect of Plasma Pretreatment on Crystalline Structures of Cotton Cellulose

**Figure 8:**
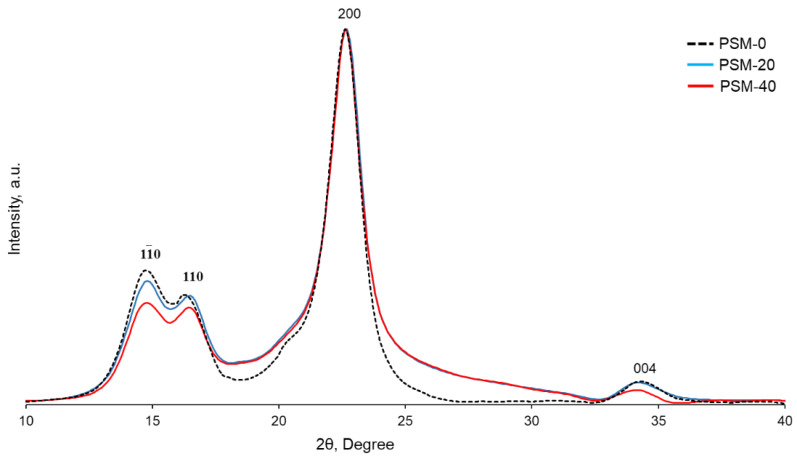
XRD patterns of control and oxygen plasma surface modified (PSM-20 and PSM-40) cotton fibers.

pXRD analysis was performed to evaluate the effect of oxygen plasma irradiation on the structural organization of cellulose. X-ray diffractograms of both control and PSM cotton fibers (Figure 8) showed diffraction peaks at $1\bar{1}0$, 110, 200, and 004, corresponding to 2θ = 14.7°, 16.5°, 22.7°, and 34.4°, respectively, in accordance with the diffraction patterns of native cellulose [44]. The crystallinity index of cellulose decreased from 65% to 56% after 20 min of plasma treatment. Plasma treatment for 20 more min did not, however, result in a further decrease in crystallinity (CrI = 54%). 

While several studies have reported that plasma treatment allowed modification of the cellulose surface without affecting its bulk properties [42,54,60], the penetration depth depends on the lifetime and energy of plasma active species and is usually a few nanometers deep [61]. In addition, it has been reported that the effect of oxygen plasma treatment on the molecular organization of cellulose depends on the duration of treatment and prolonged treatment leads to changes in the geometry of the crystalline domain and a decreased crystallinity index [42,54,60]. Thus, Calvimontes et al. reported a decrease in the crystallinity of cellulose after 8 min of microwave oxygen plasma treatment [42]. In our case, plasma treatment for 20 min resulted in the penetration of active plasma species into the cellulose fibers and affected the crystalline domain of cellulose. Yet, plasma treatment beyond 20 min did not result in further fibers’ penetration, resulting in essentially the same crystallinity. 

This study demonstrated that the dissolution of cotton cellulose in NaOH/urea system is favored by prolonged exposure to oxygen plasma. The dissolution of 1% (*w*/*v*) control cotton fibers was only 2%, while it reached ~36% and ~70% (*p* ≤ 0.05) following oxygen plasma treatment for 20 and 40 min. A similar improvement in cellulose dissolution by dielectric barrier discharge (DBD) plasma treatment was reported [53]. 

It appeared that changes in the surface structures of cotton fibers as well as the reduction in the MW and crystallinity of cellulose most likely contributed to the improved dissolution of intact cotton fibers. Surface changes improved solvent diffusion and, therefore, facilitated cellulose dissolution. It has been reported that the changes in surface morphology of cellulose resulting from plasma etching help increase its interaction with the solvent, which enhances cellulose dissolution [48,49,53]. 

In regard to MW, every glucose unit in the long polymeric chains of cellulose contributes to the formation of intra-and inter-molecular hydrogen bond network to maintain the supra-molecular structure [62]. Therefore, the freedom of conformational changes of cellulose is hindered, which is associated with the stiffness of cellulose due to the low configuration entropy in the solution [24]. However, the entropic driving force for cellulose dissolution increases rapidly with the decrease in the MW [24].

A significant improvement in the solubility of cellulose was achieved when cotton fibers were pretreated in O_2_-plasma for 20 min and 40 min. This suggests that the contact between cellulose chains and solvent molecules increased with increasing plasma pretreatment time because short cellulosic chains provide more solvent-accessible surface area [63]. However, a complete dissolution of cellulose was not achieved. It has been reported that a complete dissolution of cellulose in an aqueous solution of NaOH/urea, pre-conditioned at −12 °C, could not be achieved if the DP of cellulose is higher than 618 [62]. 

**Figure 9:**
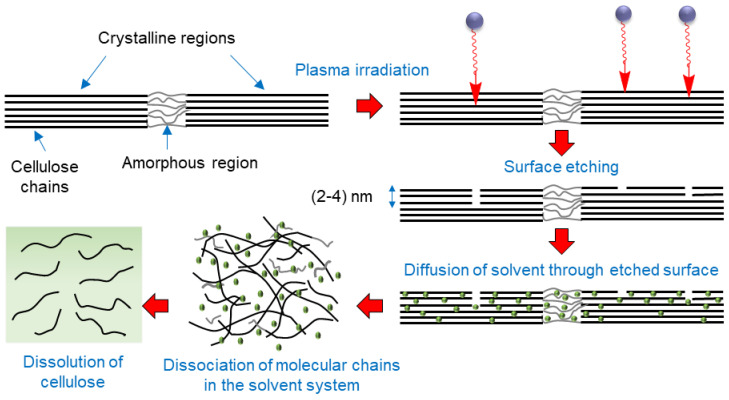
Schematic showing a possible mechanisms of enhanced cellulose dissolution induced by oxygen plasma pretreatment of cellulose.

Based on the results, we suggest the following schematic as a possible mechanism of creating a conducive environment by oxygen plasma pretreatment of cellulose for improving its dissolution (Figure 9). Surface etching of cellulose by active plasma species facilitates the diffusion of the solvent into the interfibrillar space in cellulose. It causes dissociation of molecular chains of cellulose, making them susceptible to dissolution.

## 3. Methods

### 3.1. Materials

Sodium hydroxide pellets (NaOH, ≥ 98%), Urea (NH_2_C(S)NH_2_, 99.5%), Sodium carbonate (Na_2_CO_3_, ACS certified), and Acetone (≥ 98%) were purchased from Fisher Scientific^TM^ (Fairlawn, NJ, USA). Sulfuric acid (H_2_SO_4_, ≥ 95%), Sodium silicate (Na_2_SiO_3_, reagent grade), and Triton X-100 (C_14_H_22_O(C_2_H_4_O)_n_, n = 9–10, Laboratory grade) were purchased from Sigma Aldrich (St. Louis, MO, USA). Sodium hypochlorite (NaOCl, 6% Sodium hypochlorite in water, Commercial grade) was purchased from Clorox Company (Oakland, CA, USA).

Low-quality cotton with a micronaire of 2.4 was collected from the Fiber and Biopolymer Research Institute (FBRI), Texas Tech University (Lubbock, TX, USA). Cotton fibers were purified following a protocol reported in a previous study, using scouring and bleaching chemicals, including Triton X-100, Sodium hydroxide (NaOH), Sodium hypochlorite (NaOCl), Sodium silicate (Na_2_SiO_3_), and Sodium carbonate (Na_2_CO_3_) [44].

### 3.2. Methods

#### 3.2.1. Microwave Pretreatment of Cotton Fibers

Purified cotton fibers (never ground intact fibers) were oven-dried at 105 °C overnight (Isotemp, Thermofisher Scientific, Pittsburg, PA, USA) and subsequently subjected to microwave oxygen plasma treatment using a plasma chamber (PLASMAtech Inc., Erlanger, KY, USA) (flow rate = 60 mL/min, pressure = 25 Pa, generator frequency = 2.45 GHz, time = 8 min to 48 min with an increment of 4 min, power = 500 W). Plasma surface-modified (PSM) cotton fibers were identified as PSM-0 (untreated), PSM-8 (8 min), PSM-12 (12 min), PSM-16 (16 min), etc.

#### 3.2.2. Fiber Dissolution

Plasma surface-modified cotton fibers were dissolved in NaOH/urea solvent system following the protocol reported by Cai et al. [64]. In summary, an aqueous solution of NaOH/Urea (NaOH/Urea/water = 7:12:81 by weight) was precooled to −12 °C. Cotton fiber (1% (*w*/*v*)) was added to the precooled (−12 °C) solution and stirred for 30 min while allowing the solution to warm to ambient temperature. The resultant solutions were analyzed under a polarized light microscope (PLM) (Nikon Eclipse LV 100, Nikon Corporation, Tokyo, Japan) equipped with NIS-Elements imaging platform, to evaluate the effect of plasma exposure time on the fiber dissolution. To prevent the precipitation of undissolved fiber fragments in the sample vials, the mixtures were vigorously stirred immediately before collecting the samples for microscopic observation.

Cellulose dissolution as a function of plasma treatment time was also studied using an ultraviolet-visible (UV-vis) spectrophotometer (PerkinElmer, Lambda 650, Shelton, CT, USA) by measuring the transmittance of the light through a solution of cellulose in NaOH/urea between 250 and 800 nm. The corresponding NaOH/urea solution was used as a reference for the measurement of optical transmittance of cellulose/NaOH/urea solutions. The samples were shaken immediately before measuring the transmittance, and three readings were recorded from each treatment.

#### 3.2.3. Solubility of the Plasma-Treated Cotton Fibers

The amount of cellulose dissolved as a function of plasma treatment time was calculated using the formula reported by Zhou et al. [20]. In summary, undissolved cotton fiber cellulose was isolated by centrifugation (Eppendorf, 5430 R, Barkhausenweg, Hamburg, Germany) at 9000 rpm for 45 min and subsequent washing with water and acetone. Undissolved fiber portion was vacuum dried and denoted as *w*_2_. On the other hand, the separated NaOH/Urea solution containing the dissolved fiber fraction (*w*_1_) was neutralized with sulfuric acid (2 mol L^−1^). The precipitate formed after neutralization was filtered out, washed twenty times with water, and then three times with acetone, and vacuum dried. Three independent replications were performed for each treatment, and the average solubility (*S_a_*) was calculated using the Equation (1):

(1) $$S_{a}=\left[\frac{w_{1}}{w_{1}+w_{2}}\right]\times 100\%$$

where *w*_1_ = weight of dissolved cellulose and *w*_2_ = weight of undissolved cellulose separated by centrifugation.

#### 3.2.4. Film Preparation

Aqueous solutions of both untreated cellulose and PSM in NaOH/Urea were cast into Petri dishes and subsequently gelled under ambient temperature conditions for 24 h. The gels obtained were regenerated in deionized (DI) water (AquaOne, Amarillo, TX, USA) for three days, and DI water was exchanged every 2 h. Then, regenerated cellulose films were hot-pressed at 120 °C for 3 min (SwingPress20-0403, Across International, Livingston, NJ, USA).

### 3.3. Characterization

Untreated, PSM cotton fibers, and regenerated cellulose films were conditioned in a controlled environment for 48 h (65 ± 2% relative humidity and 21 ± 1 °C) and subsequently characterized. 

#### 3.3.1. Gel Permeation Chromatography (GPC)

The MW of untreated cotton cellulose and PSM cotton cellulose was determined using Gel Permeation Chromatography (GPC) system (Viscotek TDA 305, Malvern Panalytical, Westborough, MA, USA) equipped with an autosampler (Viscotek, Model 201, Westborough, MA, USA). The cellulose solution was prepared following the protocol reported by Liyanage et al. [8]. The GPC system was calibrated using the polystyrene standards (narrow standard PS99K and broad standard PS235K). A solution of anhydrous LiCl/DMAc (5% *w*/*v*) was used as the mobile phase (flow rate of 1 mL/min). The temperature of the separation column (ViscoGEL I series column, 7.8 mm × 300 mm (Malvern Panalytical, Westborough, MA, USA, Catalog number I-MBHMW-3078)) was maintained at 50 °C, and the sample solution (100 μL) was injected for analysis. Data analysis was performed using OmniSEC GPC software (Version 5.00, Malvern Panalytical, UK). Three independent replications were performed from each treatment. 

#### 3.3.2. Scanning Electron Microscopy (SEM)

Plasma-induced morphological changes in cotton fibers and the surface morphology of cellulose films were observed using a field emission scanning electron microscope (FESEM, S/N 4300, Hitachi, Chiyoda, Tokyo, Japan). Samples were first sputter coated with iridium in 2–3 nm thickness using Quorum Q150T ES plus (Quorum Technologies Ltd., Lewes, BN, UK) for 1 min and mounted on the sample holder with carbon tape. SEM micrographs of the samples were recorded with a 5 kV accelerating voltage at different magnifications and analyzed using Quartz PCI Imaging software (https://www.quartzimaging.com/pci-microscope-imaging-software.html, accessed on 15 September 2022) (Version 8, Quartz Imaging Corp., Vancouver, BC, Canada).

#### 3.3.3. Attenuated Total Reflectance (ATR) Fourier Transform Infrared Spectroscopy (FTIR)

FTIR spectra of cotton fibers and cellulose films were collected using FTIR spectroscopy (Spectrum 400, PerkinElmer, MA, USA) equipped with a ZnSe/diamond crystal and a pressure arm. Three spectra were collected from each sample at a spectral resolution of 4 cm^−1^ and 32 co-added scans in the mid-infrared (IR) range (4000–650 cm^−1^). FTIR spectra were baseline corrected, normalized, and subsequently compared to investigate the effect of plasma treatment on the cellulose structure.

#### 3.3.4. Powder X-ray Diffraction (pXRD)

Powder X-ray diffraction patterns of cotton fibers were recorded using X-ray diffractometer (Smartlab, HD 2711 N, Rigaku, Japan) equipped with a nickel-filtered Cu- Kα radiation (λ  =  0.15406 nm) generated at 40 kV and 44 mA. Cotton fibers were placed on the sample holder and X-ray diffractograms were acquired at a scanning speed of 1 °C/min (2θ from 5° to 50°). The diffraction pattern of the empty glass slide was subtracted from the sample diffractograms, and baseline correction and normalization were performed. Then, XRD data were exported to OriginPro software (Version 2021b, OriginLab Corporation, Northampton, MA, USA) and individual crystalline peaks were extracted using the peak fitting feature of the software, assuming Gaussian function for each peak [65]. Crystallinity index (CrI) of untreated and PSM cotton fibers was calculated from the ratio of the sum of the areas under the crystalline diffraction peaks and the total area under the diffraction pattern, respectively.

#### 3.3.5. Statistical Analysis

One-way analysis of variance (ANOVA) was performed with plasma treatment time as a factor to determine the difference between means of independent variables (MW, solubility, light transmission) at 95% confidence interval using STATISTICA (Version 13.3; July 2018; TIBCO software, CA, USA).

## 4. Conclusions

The dissolution of cellulose is crucial for its transformation into high-value products. In this study, we used a plasma pretreatment for enhancing the dissolution of cellulose in NaOH/urea solvent system. We focused on the effect of microwave plasma (O_2_ gas) treatment on the surface structure of intact cotton fibers and the chemical structure of cellulose, which leads to an improved dissolution of cotton cellulose. According to FTIR results, plasma irradiation changed the surface chemistry of cellulose by depolymerizing molecular chains and forming carboxylic groups. SEM indicated the occurrence of surface etching of cotton fibers. Furthermore, XRD analysis demonstrated that the crystalline structure of cellulose was significantly reduced after 20 min of plasma treatment.

The MW of cellulose was also significantly reduced as a function of plasma exposure time likely due to the depolymerization of cellulose chains. Though the MW of cellulose has a substantial impact on the mechanical characteristics of cellulosic materials, high MW cellulose has poor processability [66]. The high molecular weight of cellulose contributes to the high mechanical properties of cellulosic materials. Low MW cellulose, on the other hand, is typically utilized in various applications that do not require high mechanical performance (e.g., disposable cellulosic products, designed for a single use, after which they are recycled or disposed of as solid waste). Furthermore, few of the studies showed that low MW cellulose helps produce high performance materials [67,68]. The benefit of plasma treatment is the ability to prepare a polymer of lower MW. For instance, synthetic and semi-synthetic (PLA) polymers are commercially available from suppliers in a range of MW: from low to high. The same is true for natural polymers, where a large number of potential applications might be realized with low MW polymer. For example, the addition of a low MW MCC has been used for reinforcement of a large range of polymeric architectures [69,70,71,72], where it was utilized as a binding filler to increase material’s rigidity [73]. Besides, since low MW cellulose stretches more easily than high MW cellulose after regeneration due to less intermolecular entanglement, it assists in the production of highly oriented spun fibers with higher tensile strength [67]. Moreover, using a high load of low MW cellulose also makes it possible to produce cellulosic materials with a higher tensile modulus [68]. Therefore, it is necessary to keep a balance between the property and the processability by tuning the MW and concentration of cellulose.

Cellulose dissolution is a heterogeneous process, which consists of solvent diffusion into the cellulose, penetration of the cellulose structure, solvation of polymeric molecules, and diffusion of solvated polymer into the solvent. In our work, we found that the effect of plasma treatment on cellulose dissolution was complex: both the solubility and dissolution kinetics were shown to be strongly dependent on its MW, crystallinity, and surface defects. In regard to MW, size of the molecule and extent of self-entanglement will affect the solvent diffusion rate. Similarly, the presence of surface defects makes fiber surface more heterogeneous allowing easier solvent penetration into fibers. Likewise, lower crystallinity favors quicker solvent diffusion into amorphous domains. As for the mechanism of cellulose dissolution, earlier studies of cellulose in aqueous NaOH/urea suggested that it involves initial formation of NaOH-hydrates, which then hydrogen-bond with cellulose macromolecule forming an inclusion complex Cellulose/NaOH/H_2_O [74]. This inclusion complex becomes surrounded by an urea shell, which separates it from other cellulosic chains preventing polymer’s aggregation. The rate of formation of such complexes also depends on the physicochemical properties of the polymer. However, the study of dissolution kinetics of plasma-treated cellulose is a research project on its own, and we plan on investigating this in the future. This study demonstrated that microwave oxygen plasma pre-treatment could be an eco-friendly and promising approach for effective dissolution of cellulose in greener solvents.

## Figures and Tables

**Table 1:** Weight averaged molecular weight (M_W_), number averaged molecular weight (M_n_), degree of polymerization (DP), and PDI of cellulose in the control (PSM-0) and plasma treated (PSM-20 and PSM-40) cotton fibers (average of three replicates).

Plasma Treatment Time (min)	Average M_W_, Da	Average Mn, Da	DP	PDI (M_W_/M_n_)
0 min (PSM-0)	394,889 ± 80,480	211,902 ± 81,973	2438 ± 497	2.09 ± 0.83
20 min (PSM-20)	253,592 ± 4198	122,151 ± 26,173	1566 ± 26	7.10 ± 5.44
40 min (PSM-40)	159,192 ± 24,634	285,844 ± 39,225	983 ± 152	3.82 ± 2.00

## Data Availability

The data presented in this study are available on request from the corresponding author.

## Sample Availability

Samples of the compounds are not available from the authors.
